# Inhibition of Sp1 prevents ER homeostasis and causes cell death by lysosomal membrane permeabilization in pancreatic cancer

**DOI:** 10.1038/s41598-017-01696-2

**Published:** 2017-05-08

**Authors:** Patricia Dauer, Vineet K. Gupta, Olivia McGinn, Alice Nomura, Nikita S. Sharma, Nivedita Arora, Bhuwan Giri, Vikas Dudeja, Ashok K. Saluja, Sulagna Banerjee

**Affiliations:** 1 Department of Pharmacology, University of Minnesota, Minneapolis, MN 55455 USA; 2 Department of Surgery, University of Miami, Miami, FL 33136 USA

## Abstract

Endoplasmic reticulum (ER) stress initiates an important mechanism for cell adaptation and survival, named the unfolded protein response (UPR). Severe or chronic/prolonged UPR can breach the threshold for survival and lead to cell death. There is a fundamental gap in knowledge on the molecular mechanism of how chronic ER stress is stimulated and leads to cell death in pancreatic ductal adenocarcinoma (PDAC). Our study shows that downregulating specificity protein 1 (Sp1), a transcription factor that is overexpressed in pancreatic cancer, activates UPR and results in chronic ER stress. In addition, downregulation of Sp1 results in its decreased binding to the ER stress response element present in the promoter region of Grp78, the master regulator of ER stress, thereby preventing homeostasis. We further show that inhibition of Sp1, as well as induction of ER stress, leads to lysosomal membrane permeabilization (LMP), a sustained accumulation of cytosolic calcium, and eventually cell death in pancreatic cancer.

## Introduction

Pancreatic cancer is the fourth leading cause of cancer deaths in the United States. It was estimated that in 2016, 53,070 people would be diagnosed with pancreatic cancer and nearly 42,000 would die from it in the United States alone^1^. There is currently no effective treatment approved for pancreatic cancer. Gemcitabine has been the standard of care for pancreatic cancer for 19 years; however, it adds a survival advantage of only 6.8 months^2^. The most widely used treatment algorithms employing gemcitabine in combination with nanoparticle bound albumin (nab-paclitaxel) or the chemo-intensive regimen of FOLFIRINOX (5-fluoruracil, oxaliplatin, and folate supplementation), offer a meager survival benefit of a few weeks over traditional gemcitabine alone regimens^3^. Preclinical studies from our laboratory have shown that triptolide, a diterpene triepoxide from a Chinese herb, is cytotoxic in a number of cancers, including pancreatic ductal adenocarcinoma (PDAC)^4^. Our laboratory has synthesized Minnelide, a water-soluble prodrug of triptolide, which just completed Phase I clinical trials^4^. Though the exact mechanism of triptolide induced cell death remains unknown, our previous studies have indicated that triptolide-induced cell death is caused by chronic Endoplasmic Reticulum (ER) stress^5^. However, there remains a gap in knowledge on the molecular mechanism of how ER stress is stimulated and how chronic ER stress causes cell death in pancreatic cancer.

The ER is a site for calcium storage, protein folding, and modifications^6–10^. ER stress is a well-characterized condition that plays a role in multiple pathologies, including neuro-degeneration, rheumatoid arthritis, atherosclerosis, and many types of cancer^10, 11^. A variety of cellular insults can result in ER stress, including hypoxia, calcium fluctuations, oxidative stress, and nutrient deprivation^8–10^. When ER function is impaired during stress, the cell initiates the evolutionarily conserved unfolded protein response (UPR), which helps to adapt and survive stressful conditions. The UPR is mediated by three transmembrane sensors, inositol requiring enzyme 1 (IRE1), activating transcription factor 6 (ATF6), and protein kinase-like ER kinase (PERK). During normal conditions, these three sensors are kept silent by associating with glucose regulatory protein 78 (Grp78), a protein chaperone. Upon experiencing stress, Grp78 will disassociate from the sensors to activate the UPR. In cases of severe or chronic/prolonged ER stress, the UPR is overwhelmed, and the cell will initiate cell death pathways^8^. In some diseases like neuro-degeneration or type II diabetes, recent studies show promise in inhibiting ER stress, resulting in cell survival^11^. However, in cancer, treatment strategies aim to prevent ER homeostasis to cause cell death^11^.

Multiple studies have underscored the importance of Specificity protein 1 (Sp1), a transcription factor, in malignant tissues. In fact, Sp1 has recently been described as a non-oncogene addiction gene in cancer^12^. Sp1 has been reported to regulate many biological functions, including cell growth, differentiation, survival, tumor progression, and metastasis^12–18^. It has also been reported in colon, gastric, pancreatic, and breast cancers that Sp1 is overexpressed, whereas minimal to no Sp1 expression is detected in normal differentiated cells^12–17^. Owing to its binding sites present in a large number of genes, downregulation of Sp1 is likely to have a profound effect on cancer cell survival. Further, in the context of ER stress, it has been reported by Safe *et al*., that Sp1 binding is necessary for the UPR^19^. However, it has not been determined whether chronic ER stress as a result of Sp1 downregulation leads to cell death in pancreatic cancer.

One of the hallmarks of cancer cells is their resistance to apoptosis. Thus, a number of studies have been focused on overcoming this resistance by targeting pathways leading to apoptosis. Lysosomal membrane permeabilization (LMP) often precedes apoptosis in response to cytotoxic compounds in cancer cells^20, 21^. Lysosomes contain a number of hydrolytic enzymes, and are essential in autophagic-mediated recycling and cell survival^20, 21^. Due to the contents of lysosomes, they have been described as “suicide bags”, and when permeabilized, the contents can be released into the cytosol, leading to unregulated proteolysis and cell death^20^.

Calcium regulation is also an important component of cell survival and thus, cell death^6, 7, 22^. Calcium is also a second messenger to a variety of cellular processes, including protein synthesis and folding, proliferation, and apoptosis^6, 23^. The ER and mitochondria provide the major storages sites of calcium for the cell, and a dysregulation of calcium can not only cause ER stress, but can also be a consequence of ER stress, and lead to cell death^7, 22, 24^.

Previous studies from our laboratory have shown that triptolide results in chronic ER stress by downregulating glucose regulatory protein 78 (Grp78), leading to cell death in pancreatic cancer^5^. In addition to these effects, triptolide downregulates the activity of Sp1^13^, thereby starting a cascade of events that lead to downregulation of several pro-survival genes. In this study, we show that Sp1 downregulation in pancreatic cancer results in chronic ER stress, which in turn leads to a sustained increase in cytosolic calcium, LMP, and cell death.

## Results

### Sp1 downregulation causes endoplasmic reticulum stress and tumor regression

**Figure 1:**
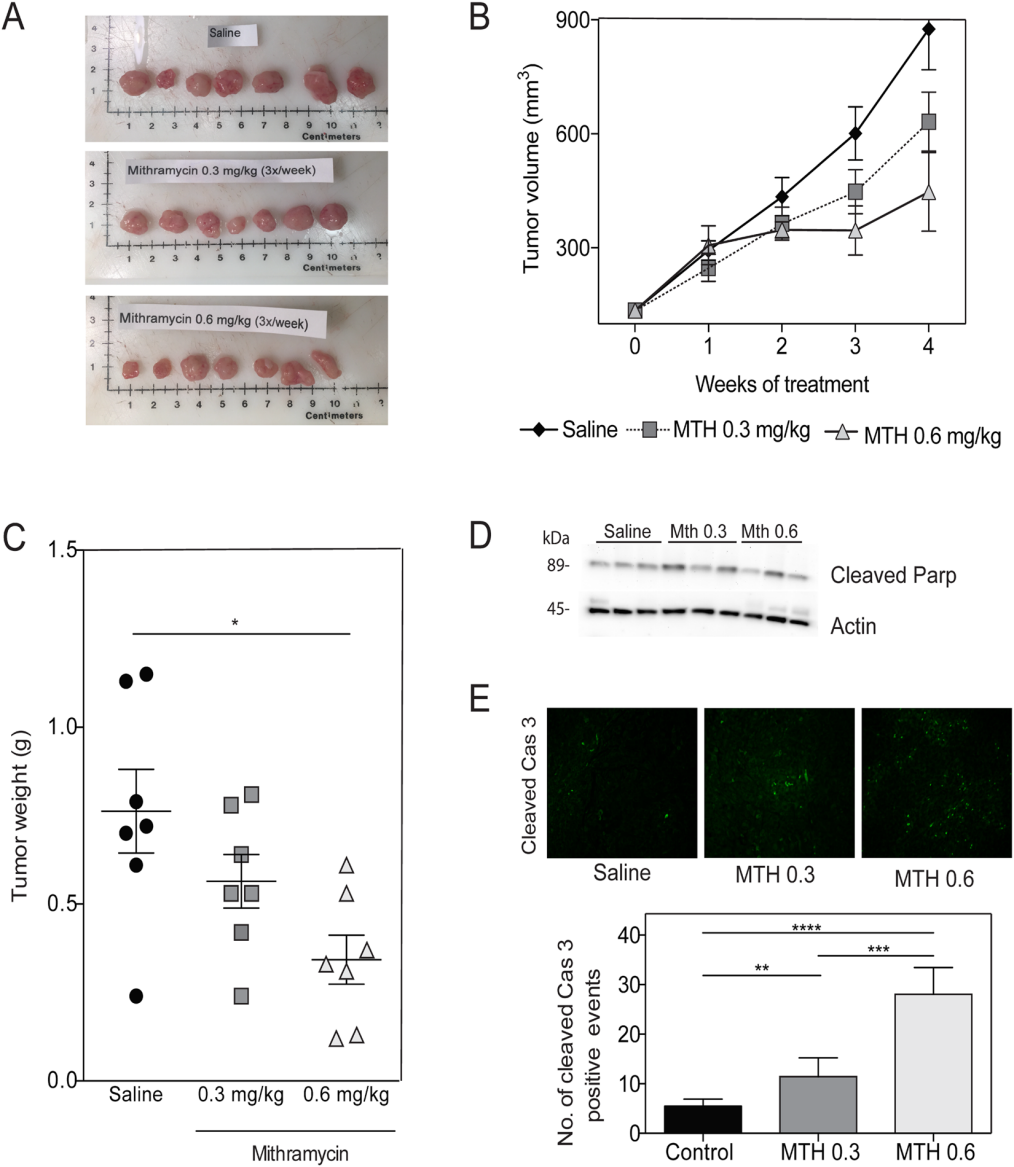
Sp1 downregulation causes tumor regression. MIA PaCa-2 cells injected subcutaneously, and treated with saline or mithramycin for 4 weeks. (**A**) Tumors explanted from saline, mithramycin 0.3 mg/kg, and mithramycin 0.6 mg/kg (**B**) Tumor volume (**C**) Tumor weights (**D**) Tumors analyzed for apoptosis with cleaved PARP by western blot, and (**E**) cleaved caspase 3 by immunofluorescence.

We have previously shown that Sp1 downregulation with mithramycin (MTH) results in pancreatic cancer cell death^13^. In the current study, we show that both 0.3 and 0.6 mg/kg MTH decrease tumor burden in a subcutaneous model with MIA PaCa-2 cells in athymic nude mice (Figure 1A), which is consistent with our previous work. After four weeks, the average tumor volume in saline treated mice was 876 mm^3^, whereas mice treated with 0.3 mg/kg MTH and 0.6 mg/kg of MTH was 632 mm^3^ and 447 mm^3^ respectively (Figure 1B). The average tumor weights in the MTH treated mice were also decreased (0.56 g in 0.3 mg/kg; 0.34 g in 0.6 mg/kg) compared to saline treated mice (0.76 g) (Figure 1C). Inhibition of Sp1 also led to an increase in PARP cleavage (Figure 1D) and increased cleaved caspase 3 staining (Figure 1E), indicating apoptotic cell death in these tumors.

Consistent with our findings *in vivo*, treatment with MTH also caused UPR in pancreatic cancer cell lines MIA PaCa-2 and S2-VP10 and S2-013 (Supplementary Figure 1A,B). We further observed that while a short treatment (6 hours) with MTH elicited an “acute” response by triggering the unfolded protein response (UPR) in the cancer cells, a longer treatment (24 hours) with MTH caused a complete breakdown of the ER stress machinery resulting in downregulation of UPR genes and causing “chronic” ER stress (Supplementary Figure 1B). Further, upon silencing Sp1 in these cells using siRNA, we observed a similar induction of chronic ER stress in 24–48 hours (Supplementary Figure 2A,B).

### Sp1 downregulation leads to chronic endoplasmic reticulum stress via homeostatic disruption

**Figure 2:**
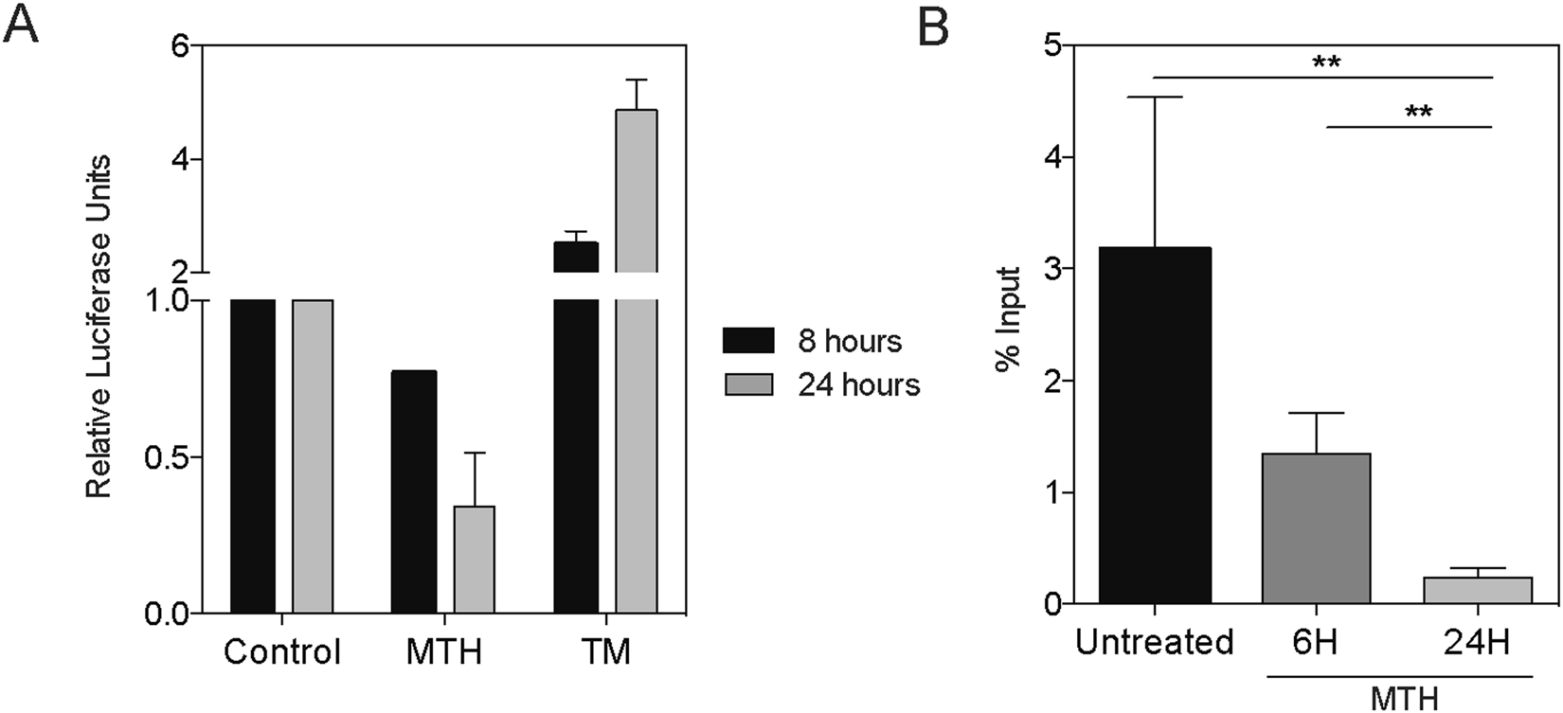
Sp1 downregulation leads to chronic ER stress by deregulating the homeostatic mechanism. Mithramycin treated MIA PaCa-2 cells analyzed for (**A**) ERSE dual-luciferase activity assay from 0–24 hours, and (**B**) ChIP of Sp1 on the Grp78 promoter, from 0–24 hours.

In order to elucidate how MTH causes chronic ER stress, we focused on the “master regulator” of UPR, Grp78. We first performed an activity assay of ER Stress Response Elements (ERSE), using a Cignal reporter assay (Qiagen). ERSE are the binding motifs that transcriptionally regulate UPR-induced genes. It has been reported that NF-Y, YY1, and Sp family proteins, along with multiple ER stress-associated transcription factors all bind to the ERSE during a stress response^19^. Upon treatment with 100 nM MTH, the ERSE activity decreased to 0.771 RLU (relative luciferase units) compared to untreated cells (normalized to 1.0) in 8 hours (Figure 2A). After 24 hours of treatment, MTH treated cells showed a further decreased activity of 0.344 RLU compared to untreated cells (normalized to 1.0). Tunicamycin, a known ER stress inducer, was used as a positive control, and showed a robust increase in ERSE activity compared to untreated cells in 8 and 24 hours (2.500 and 4.861 RLU, respectively).

To study if the decrease in ERSE activity was owing to decreased binding of Sp1 to the Grp78 promoter elements, we performed chromatin immunoprecipitation of Sp1 on the Grp78 promoter. MIA PaCa-2 cells treated with 100 nM MTH showed a decrease in the % input of Sp1 on the Grp78 promoter. Untreated cells had a % input of 3.177, whereas 6 hours of MTH treatment decreased to 1.348% and 0.239% in 24 hours (Figure  2B).

### ER stress initiates cell death via lysosomal membrane permeabilization

**Figure 3:**
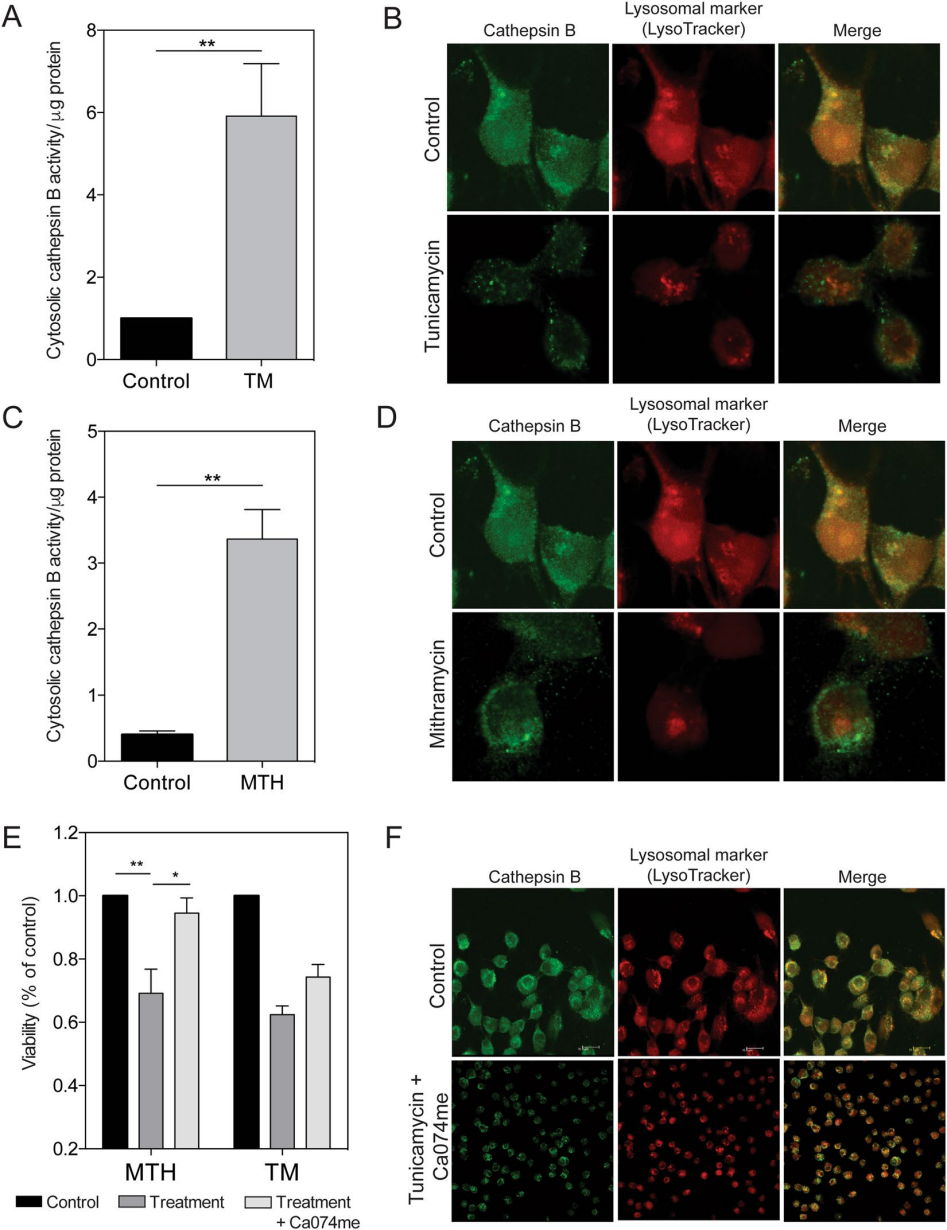
ER stress leads to a disruption in the lysosomal membrane. MIA PaCa-2 cells analyzed for lysosome membrane permeabilization. (**A**) Cells treated with 1 μM tunicamycin and analyzed for cytosolic cathepsin B activity. (**B**) Cells treated with 1 μM tunicamycin, and stained with cathepsin B and lysotracker. Images taken at 60x magnification. (**C**) Cells treated with 100 nM mithramycin and analyzed for cytosolic cathepsin B activity. (**D**) Cells treated with 100 nM mithramycin, and stained with cathepsin B and lysotracker. Images taken at 60x magnification. (**E**) Viability determined for cells treated with 100 nM mithramycin or 1 μM tunicamycin, with and without a cathepsin B inhibitor, Ca074me. (**F**) Cells treated with 1 μM tunicamycin, with and without Ca074me detected by immunofluorescence. Images taken at 40x and 60x magnification.

To characterize how chronic ER stress, mediated by downregulation of Sp1, results in cell death, we examined two common processes associated with apoptosis: permeabilization of lysosomal and mitochondrial membranes. We first studied the effect of chronic ER stress on lysosomal integrity. Lysosomal membrane permeabilization leads to a release of cathepsins (lysosomal enzymes) into the cytosol. Treatment for 1 hour with 1 μM tunicamycin (TM), a known ER stress inducer, resulted in a 5.92-fold increase in cathepsin B cytosolic activity in MIA PaCa-2 cells compared to untreated cells (Figure  3A). By downregulating Sp1 with MTH for 1 hour, cathepsin B activity was 3.36-fold greater than untreated MIA PaCa-2 cells (Figure 3C). Additionally, using immunofluorescence detection, we saw a release of cathepsin B from the lysosomes upon treatment with TM (Figure 3B), thapsigargin (TG) (Supplementary Figure 4A), brefeldin A (BFA) (Supplementary Figure 4B), as well as MTH (Figure 3D) for 6 hours. These findings unequivocally show that induction of chronic ER stress leads to lysosomal membrane permeabilization, and release of cathepsin B into the cytosol. Further, pre-treatment with a cathepsin B inhibitor, Ca074me, results in a rescue of viability after 24 hours (Figure 3E). MIA PaCa-2 cells were also treated for 6 hours with tunicamycin and a cathepsin B inhibitor, which resulted in a reduction of cathepsin B release from the lysosomes compared to tunicamycin alone, as detected using immunofluorescence (Figure 3F).

Since mitochondrial outer membrane permeabilization (MOMP) is also a commonly reported method of cell death, we sought to see if MOMP was another mechanism through which chronic ER stress led to cell death. MIA PaCa-2 cells were treated for 1 and 6 hours with TM, TG, BFA, and MTH, and assayed for disruption of mitochondrial membrane potential (MMP). Based on our results, we can conclude that while TM had a minor disruption in MMP compared to the positive control, FCCP, the other inhibitors did not affect MMP within 6 hours of treatment (data not shown).

### Chronic ER stress results in a sustained accumulation of cytosolic calcium

**Figure 4:**
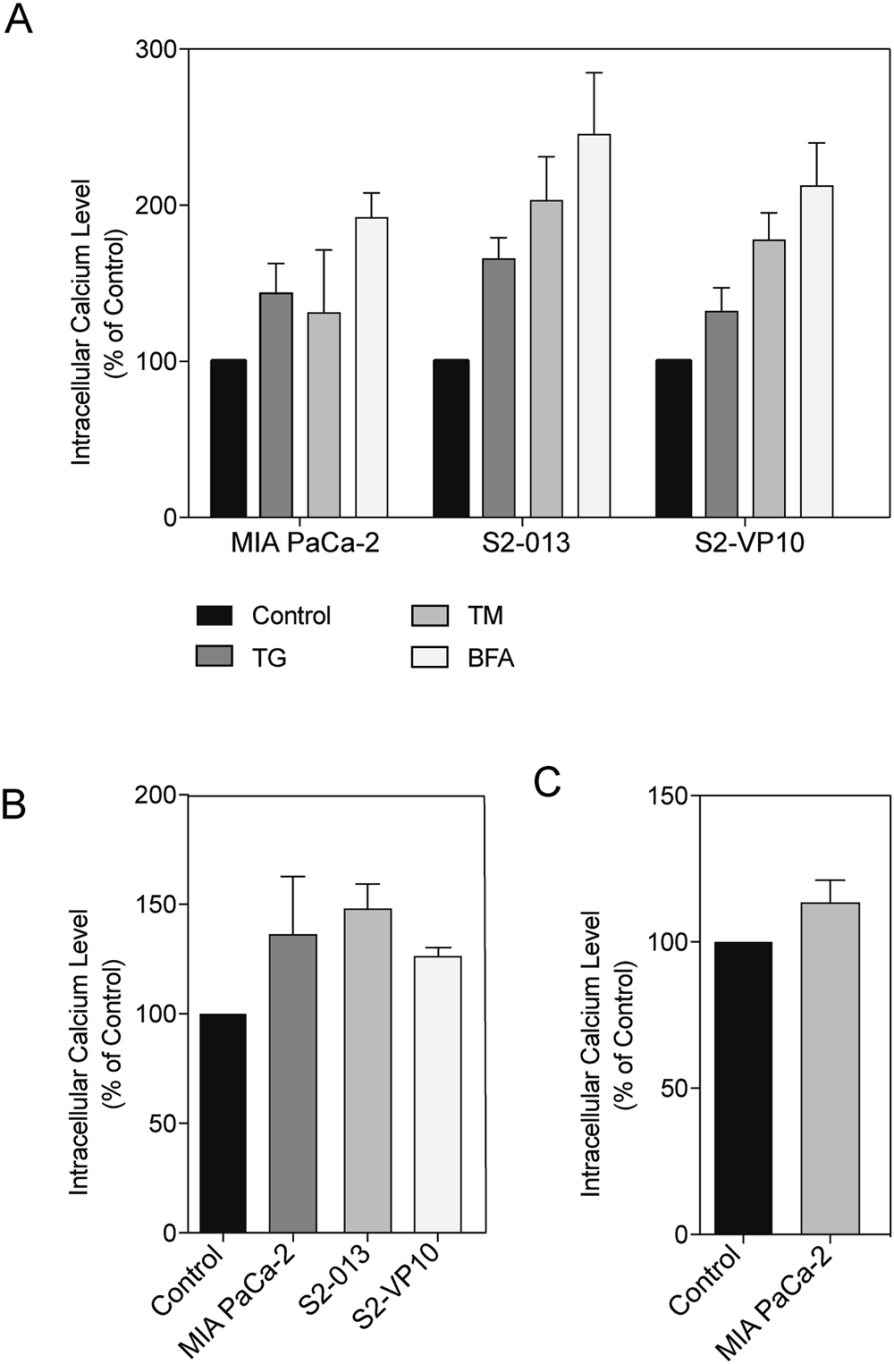
Chronic ER stress results in a sustained accumulation of cytosolic calcium. MIA PaCa-2, S2-VP10, and S2-013 cells treated with (**A**) known ER stress inducers and measured cytosolic calcium after 24 hours of treatment, and (**B**) mithramycin and measured cytosolic calcium after 24 hours of treatment, and (**C**) silenced with siSp1 measured cytosolic calcium after 24 hours of transfection.

Calcium imbalance in the cytosol is a known inducer of ER stress in a cell, as calcium is one of the co-factors needed for protein folding. Sustained calcium release from the calcium stores in the cellular organelles, in response to a drug or noxious stimuli, eventually leads to cell death. Therefore, we measured the amount of cytosolic calcium in cells treated with multiple pharmacological inhibitors simulating chronic ER stress. Treatment of MIA PaCa-2, S2-VP10, and S2-013 cells with 100 nM MTH, 100 nM TG, 1 μM TM, and 1 μM BFA resulted in a sustained accumulation of cytosolic calcium between 6–24 hours, measured using calcium orange dye based assay method (Figure 4), as well as Fura-2 (data not shown). While 1 hour did not result in an appreciable amount of cytosolic calcium (data not shown), by 24 hours, we observed a sustained calcium accumulation in the cytosol. MIA PaCa-2 cells treated with Sp1 siRNA and pharmacological inhibitors showed the following calcium levels at 24 hours: untreated 100%, TG 143.5%, TM 131.0%, BFA 192.0% (Figure 4A), MTH 136.0% (Figure 4B), siSp1 113.2% (Figure 4C). S2-VP10 cells treated with pharmacological inhibitors showed the following calcium levels at 24 hours: untreated 100%, TG 131.9%, TM 177.6%, BFA 212.4% (Figure 4A), MTH 126.0% (Figure 4B). S2-013 cells treated with pharmacological inhibitors showed the following calcium levels at 24 hours: untreated 100%, TG 165.5%, TM 203.0%, BFA 245.3% (Figure 4A), MTH 147.7% (Figure 4B). To determine if sustained accumulation of calcium was responsible for the cell death, we next used 10 μM of BAPTA (a calcium chelator) with each pharmacological inhibitor (data not shown). While there was a decrease in cytosolic calcium upon treatment with BAPTA, the viability of the cells was unaffected (data not shown). This finding suggests that even though induction of chronic ER stress led to an accumulation of cytosolic calcium at 24 hours, cytosolic calcium did not precipitate the UPR.

## Discussion

ER stress is a “condition” due to an accumulation of unfolded proteins in the ER. A variety of cellular insults can result in the accumulation of unfolded or incorrectly folded proteins^8–10^. In response to ER stress, a cell can initiate the UPR to adapt and survive, thereby re-establishing ER homeostasis. However, severe or chronic UPR can breach the threshold for survival, and lead to cell death. ER stress is an important mechanism, which has been implicated in various pathologies, and has the potential to be targeted therapeutically^10, 11, 25, 26^. There have been prior studies which have described UPR as a druggable mechanism in cancer because of the distinct metabolic requirements of cancer cells, and the activity of UPR compared to normal cells^25, 26^. As a result, it is important to understand how this mechanism works in cancer to find druggable targets, which can preferentially induce cancer cell death in cancer cells, while avoiding cellular adaptation and survival.

Previous studies have determined that Sp1 is necessary for an ER stress response^19^, and cancer cell survival^12, 13^. In order to restore ER homeostasis, an active Grp78 transcription is required. Interestingly, the RNA and protein expression of Grp78 remained relatively unchanged, as the cells failed to regain homeostasis following Sp1 downregulation. Based on our data from the current investigation, in the absence of Sp1, there is decreased binding of Sp1 to the Grp78 promoter (Figure 2A). Without an active transcription of Grp78, there is a breach in the threshold of stress that cells can handle, leading to cell death. By downregulating Sp1 (Figures 1 and 2), we see a decrease in viability, in ERSE activity, and binding to the Grp78 and ATF6 promoters (ATF6 data not shown); whereas, tunicamycin did not affect Sp1 binding (Supplementary Figure 3). An increase in ERSE activity is one necessary step for a cell to re-establish homeostasis. Without Sp1, Grp78 is not transcribed, and is unable to help the cells recover from ER stress, and silence the activated UPR.

In previous studies, we have shown that triptolide works in two ways 1) it downregulates the substrate pool of O-GlcNAc, the modification of Sp1, rendering it inactive; and 2) by causing chronic ER stress and cell death^5, 13, 27^. In our current study, we have shown that Sp1 downregulation also causes chronic ER stress. To further characterize the cell death mechanism induced by chronic ER stress, we studied lysosomal and mitochondrial cell death. Depending on the type of ER stress inducer, as well as the cell type, cancer cells can die by caspase-dependent or independent pathways upon LMP^20^. In AML and human monocytic leukemia/lymphoma, it has been shown that tunicamycin causes lysosome-mediated cell death, independent of the mitochondria^8^. In pancreatic cancer, bortezomib, a proteasome inhibitor, induces both LMP and MOMP^28^. Our group has published that triptolide results in LMP-mediated apoptosis, independent of MOMP^29^. However, triptolide in combination with TRAIL induces LMP and MOMP^30^. In the current study, we have shown that multiple ER stress inducers can result in LMP in PDAC (Figure  3), and initiate cell death, independent of MOMP.

To further explore ER stress-induced cell death, we studied the effect of ER stress inducers on cytosolic calcium. Calcium regulation is essential for survival of every cell. The ER is a major storage site for calcium, which can be taken up via the SERCA pump. Conversely, calcium can be released from the ER, for example, to aid in cytosolic protein folding. Further, in autophagy, calcium is necessary for the fusion of the autophagosome and lysosome^31^. Our results clearly show that acute UPR observed in the first 6 hours, initiated by either known ER stress inducers or Sp1 downregulation, does not lead to an accumulation of cytosolic calcium. However, upon onset of chronic/prolonged ER stress, there is an accumulation of calcium in the cell that leads to cell death in 24 hours (Figure 4).

**Figure 5:**
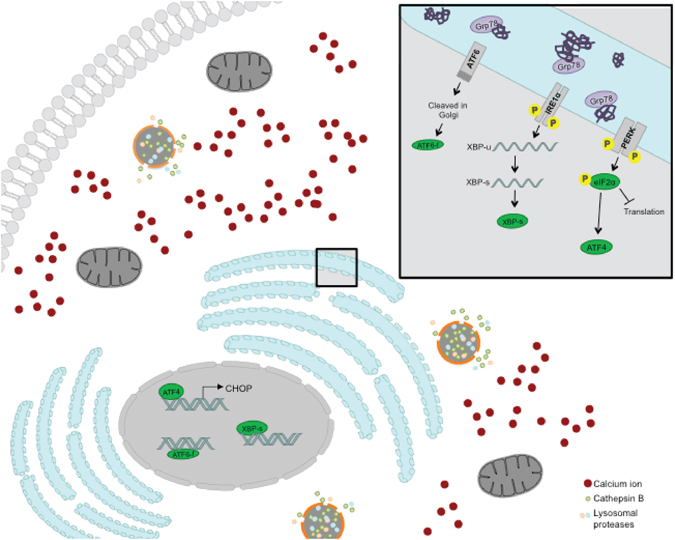
Schematic diagram showing how Sp1 downregulation prevents ER homeostasis.

Thus to summarize our results, Sp1 downregulation leads to chronic ER stress, a sustained accumulation of calcium, LMP, and cell death by preventing the ER homeostasis (Figure  5).

This study underscores the importance of ER stress and understanding the complex balance of adaptation vs. cell death in pancreatic cancer. The current study identified Sp1 and Grp78 as potential targets for future therapeutics in PDAC; discovered a protective role of Sp1 in cancer; as well as further detailed ER stress response in cell death mechanisms.

## Methods and Materials

### Cell culture and treatment

Pancreatic cancer cell line, MIA PaCa-2 (obtained from ATCC) was cultured in DMEM; high glucose, supplemented with 10% FBS and 100 units/mL penicillin and 100 μg/mL streptomycin. S2-VP10 and S2-013 cell lines (a gift from Dr. Masato Yamamoto’s lab) were grown and propagated in RPMI, supplemented with 10% FBS, 100 units/mL penicillin and 100 μg/mL streptomycin. All cells were maintained at 37 °C in a humidified air atmosphere with 5% CO_2_.

ON-TARGETplus SMARTpool human Sp1 and Grp78 siRNA (Dharmacon) were used to silence expression of the respective genes in MIA PaCa-2 cells. Pool of 4 siRNA was used for all the above genes. Transfections were completed using DharmaFECT (Dharmacon) according to manufacturer’s instructions. The following drugs were used for this study: mithramycin, tunicamycin, thapsigargin, brefeldin A (all from Sigma).

### Cell viability assay

MIA PaCa-2 cells were seeded in a 96 well plate (7,000 cells/well) and allowed to adhere for 24 hours. Cells were transfected with 20 nM siRNA or treated with 100 nM mithramycin. Cell viability assays following siRNA or pharmacological inhibitors were performed using a WST-8 based cell cytotoxicity assay per the manufacturer’s protocol (Dojindo) and expressed after normalizing to untreated cells.

### Gene expression analyses

#### RT-PCR

RNA was isolated from the cells according to manufacturer’s instructions using Trizol (Invitrogen). Total RNA (2 μg) was used to perform real-time PCR using the Quantitect SyBr green PCR kit (Qiagen) according to the manufacturer’s instructions using Roche 480 real-time PCR system. All data were normalized to the housekeeping gene 18S (18s Quantitect Primer Assay; Qiagen). Quantitative RT-PCR for Sp1, Grp78 (Qiagen).

Synthesized by Invitrogen/life technologies

ATF6 F: GGAGTATTTTGTCCGCCTGC/R: ACTGGGCTATTCGCTGAAGG

XBP-u F: CAGACTACGTGCACCTCTGC/R: GGCTGGTAAGGAACTGGGTC

XBP-s F: CTGAGTCCGCAGCAGGTG/R: GGCTGGTAAGGAACTGGGTC

CHOP F: AGATGAGCGGGTGGCAGCGA/R: CCAGGCTTCCAGCTCCCAGC

#### Western blotting

Proteins from treated and untreated cell lysates were estimated using the BCA protein estimation assay (Thermo Scientific). Blots were probed for: anti-Sp1; anti-BiP; anti-Ire1α; anti-PERK; anti-phospho-PERK (Thr980); anti-eIF2α; anti-phospho-eIF2α; anti-β-actin (all from Cell Signaling).

### Luciferase reporter assay for ERSE

MIA PaCa-2 cells were seeded in a 24 well plate. Non-silencing and siSp1 were transfected in four wells each, and allowed to incubate for 15 hours. Cells were then transfected with the cignal reporter plasmids for ERSE (Qiagen) and treated with 100 nM MTH and 1 μM TM for 8 and 24 hours. At each time point, wells were washed with PBS, and 100 μL of passive lysis buffer was added per well. After 15 minutes of rocking in passive lysis buffer, plates were stored at −80 °C until ready to read. The dual luciferase kit (Promega) was used to measure activity using a luminometer. Each sample was treated in duplicate for each plasmid (duplicates for the negative reporter and duplicates for the ERSE reporter).

### Immunofluorescence

MIA PaCa-2 cells were plated in chamber slides and incubated for 1–24 hours at 37 °C. The slides were treated with 100 nM MTH, 1 μM TM, 100 nM TG, or BFA 1 μM; fixed with 2% paraformaldehyde, and permeabilized with CHAPS. The slides were incubated with 1:1000 dilution of rabbit polyclonal anti-cathepsin B antibody (Sigma) and a 1:1000 dilution of Alexa 488-conjugated donkey anti-mouse IgG (Molecular Probes) for cathepsin B staining. The slides were mounted using Prolong Gold anti-fade with 4′,6-diamidino-2-phenylindole (Molecular Probes). Immunofluorescence images were obtained on a confocal microscope (Nikon Eclipse Ti) with a 40–100x oil-immersion objective. EZ-C software was used to obtain images.

### Chromatin immunoprecipitation

MIA PaCa-2 cells were treated with 100 nM MTH for 6 and 24 hours. Samples were collected and processed per the manufacturer’s instructions (Pierce). Grp78 ChIP primer (GPH1026964(−) 01 A; Qiagen) was used.

### Calcium measurement: calcium orange-AM

Cells were seeded in a white 96-well plate, and allowed to attach for 24 hours at 37 °C, and then treated with various ER stress inducers, including MTH and siSp1. After 24 hours of treatment, media was removed, cells were washed with phosphate-buffered saline (PBS), and loaded with 1.5 μL of 5 mmol/L Calcium Orange-AM (in 20% pluronic acid in DMSO) in serum free media for 1 hour. After incubation, the cells were washed with PBS three times, and fresh PBS was added for measurement of calcium. Luminescence measurements were obtained at 550 nM. A clear 96-well plate was used to determine viability, and the results were normalized to viability.

### Cathepsin B assay

Cells were seeded in a 6-well plate, and allowed to adhere for 24 hours. Cells were treated with 1 μM TM and 100 nM MTH for 1 hour each. To measure cytosolic cathepsin B activity, the cytosolic fraction was isolated using a cytosolic buffer (25 mM HEPES, 120 mM KCl, 0.15 mM CaCl_2_, 10 mM K_2_HPO_4_, 5 mM MgCl_2_, 2 mM EDTA, 2 mM ATP, 10 mM DTT, pH 7.6) containing 20 μg/mL Streptolysin O for 20 minutes on ice. Cells were then washed without Streptolysin O, and incubated at 37 °C for 20 minutes to selectively permeabilize the plasma membrane. The cells were then centrifuged at high speed to separate the cells and cytosol. Cathepsin B activity was determined using a cathepsin B selective substrate, N-carbobenzoxy-arginyl-arginine-naphthylamide (Bachem), as described by McDonald and Ellis^32^. Activity was expressed as units/mg protein in each sample.

### *In vivo* study

Athymic nude mice were injected with 10^6^ MIA PaCa-2 cells suspended in Matrigel (Corning), subcutaneously in the right flank. Tumor size was measured weekly and mice were randomized when tumors reached an average of 250 mm^3^. Mice were randomized into saline, MTH 0.3 mg/kg, and MTH 0.6 mg/kg groups. MTH was administered intraperitoneally, three times per week. Mice were sacrificed when tumors reached 900 mm^3^.

### Ethics Statement

All procedures were approved and performed in accordance with the relevant guidelines and regulations of University of Minnesota Institutional Animal Care and Use Committee (IACUC).

### Statistical Analysis

Values are expressed as the mean +/− SEM. All *in vitro* experiments were performed at least three times. The significance between any two samples was analyzed by t-test, values of p < 0.05 were considered statistically significant.

## Electronic supplementary material

Supplementary figures and legends
