# Supplementary material for: Inhibition of Sp1 prevents ER homeostasis and causes cell death by lysosomal membrane permeabilization in pancreatic cancer
Source: Sci Rep. 2017 May 8;7:1564. doi: 10.1038/s41598-017-01696-2 (PMC5431512; doi:10.1038/s41598-017-01696-2)

Inhibition of Sp1 prevents ER homeostasis and causes cell death by lysosomal membrane permeabilization in pancreatic cancer.

Authors: Patricia Dauer[1], Vineet K. Gupta[2], Olivia McGinn[1], Alice Nomura[1,2], Nikita S. Sharma[2], Nivedita Arora[1], Bhuwan Giri[2], Vikas Dudeja[2], Ashok K Saluja[1,2], Sulagna Banerjee*[2]

Supplementary Figure Legends

Supplementary Figure 1: **Mithramycin treatment activates the unfolded protein response**. A) MIA PaCa-2 cells treated with 100 nM mithramycin for 1-6 hours. B) S2-VP10 and S2-013 cells treated with 100 nM mithramycin for 0-24 hours. C) Protein expression of UPR-related genes in MIA PaCa-2 cells treated with 100 nM mithramycin for 0-24 hours. D) RNA splicing of XBP gene in MIA PaCa-2 cells treated with 100 nM mithramycin for 0-24 hours, compared to 18s.

Supplementary Figure 2: **siSp1 results in chronic ER stress**. MIA PaCa-2 cells transfected with siSp1 A) RNA expression of UPR related genes at 24-48 hours. B) Results in RNA splicing of XBP gene compared to 18s.

Supplementary Figure 3: **Tunicamycin does not affect Grp78 promoter binding**. MIA PaCa-2 cells analyzed for ChIP of Sp1 on the Grp78 promoter, after 6 hours of treatment with tunicamycin compared to untreated cells.

Supplementary Figure 4: **Other known ER stress inducers also result in LMP**. MIA PaCa-2 cells analyzed by immunofluorescence of cathepsin B and lysotracker after 6 hours of treatment with A) 100 nM thapsigargin B) 1 μM brefeldin A (BFA).

Supplementary Figure 1:

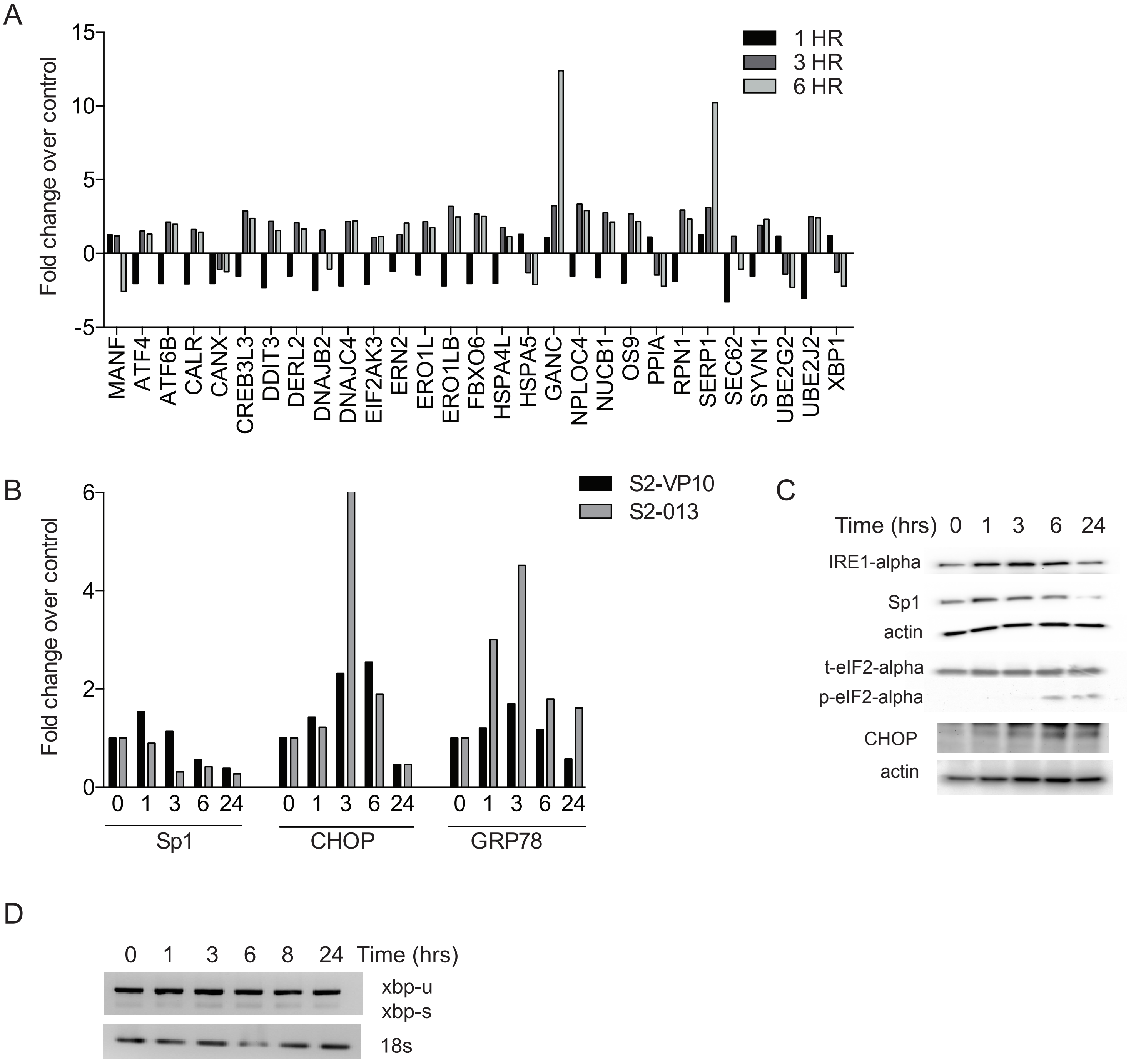

Supplementary Figure 2:

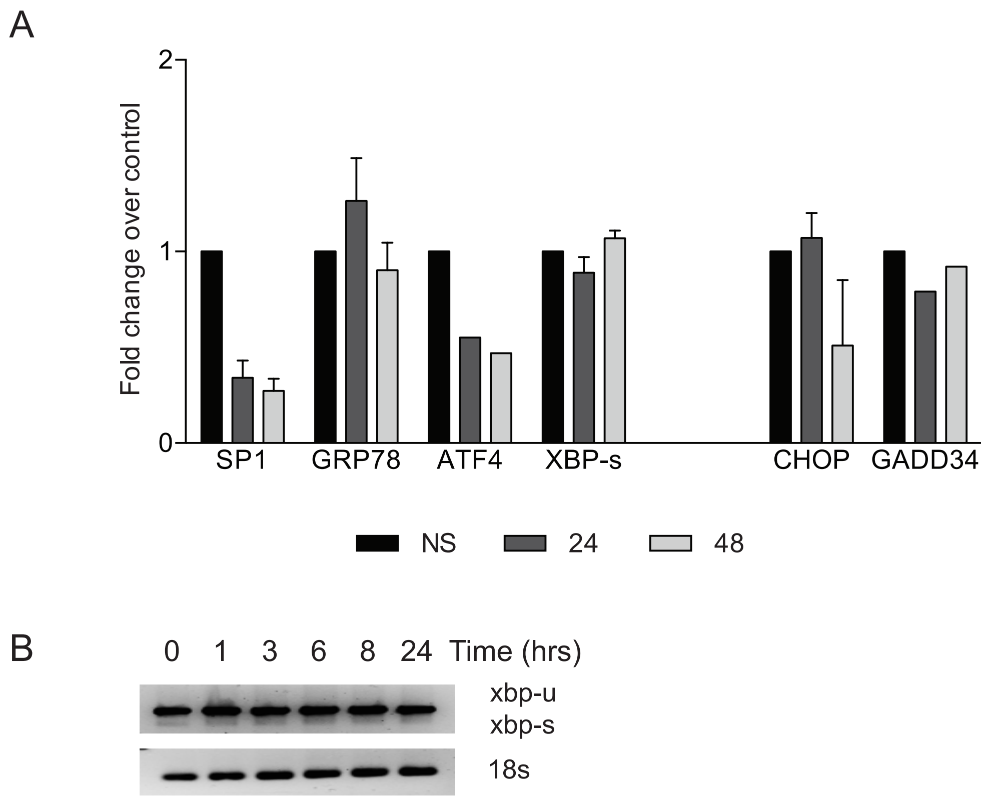

Supplementary Figure 3:

A

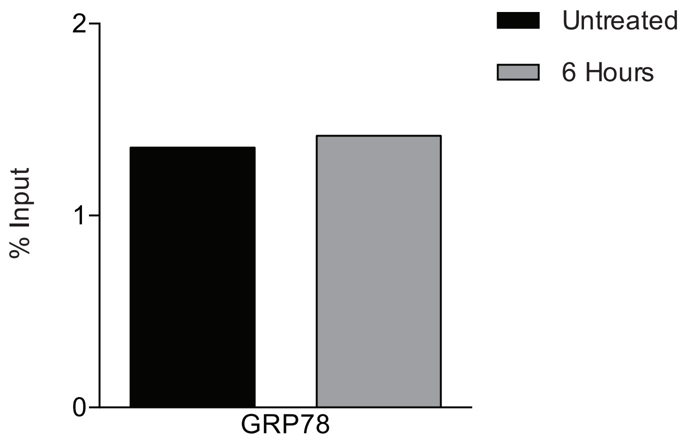

Supplementary Figure 4:

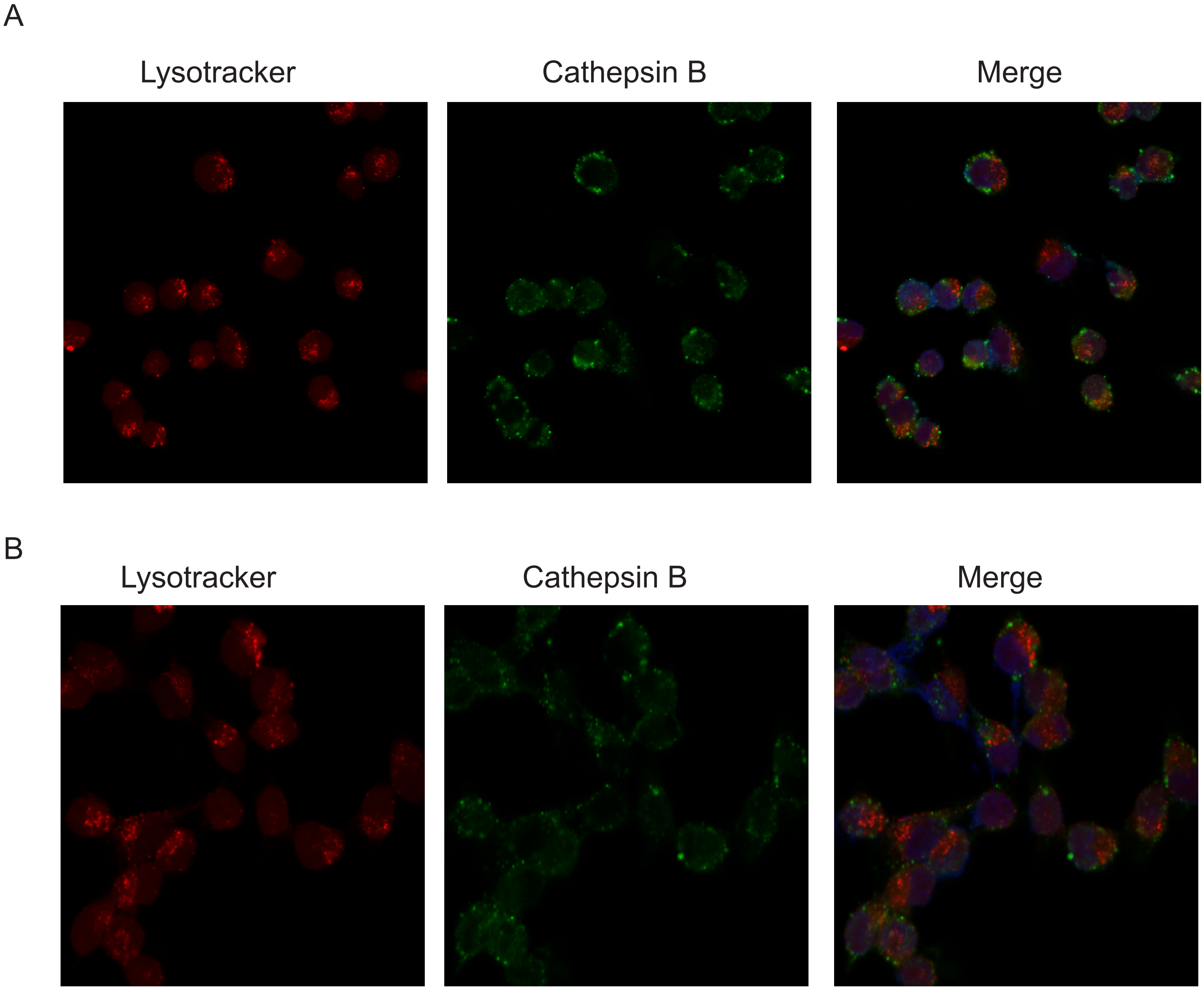

Supplement: Supplementary file 1 — Supplementary figures and legends [file 41598_2017_1696_MOESM1_ESM.pdf]
